# C–C chemokine receptor 5 and acute graft‐versus‐host disease

**DOI:** 10.1002/iid3.687

**Published:** 2022-08-17

**Authors:** Jing Yuan, Han‐yun Ren

**Affiliations:** ^1^ Department of Hematology The Second Hospital of Hebei Medical University Shijiazhuang Hebei China; ^2^ Department of Hematology Peking University First Hospital Beijing China

**Keywords:** acute graft‐versus‐host disease, C–C chemokine receptor 5, immune mechanism

## Abstract

**Background:**

The C–C chemokine receptor 5 (CCR5) is mainly expressed in a variety of immune cells. It interacts with multiple chemokine ligands that mediate the trafficking and recruitment of effector cells toward sites of inflammation. CCR5 not only plays a critical role in cell growth, activation, differentiation, adhesion, and migration but also participates in the development of acute graft‐versus‐host disease (GVHD) after allogeneic hematopoietic cell transplantation.

**Methods:**

This is a literature review article. The research design method is an evidence‐based rapid review. The present discourse aim is first to scrutinize and assess the available literature on CCR5 and acute GVHD. Standard literature and database searches were implemented, gathered relevant material, and extracted information was then assessed.

**Results:**

CCR5 is a marker of GVHD effector cells, and CCR5 expression is elevated when acute GVHD occurs. CCR5 blockade with maraviroc in clinical trials results in a low incidence of acute GVHD. The immune mechanism includes that CCR5 blockade inhibits donor T cell migration and recruitment toward target organs, reduces the absolute numbers of donor T cells, is capable of slightly suppressing dendritic cell maturation, and reduces the percentage of Th1 and Th17 subsets. CCR5 blockade also inhibits internalization and activation of chemokines, inhibits proliferation and chemotaxis of T cells, and decreases the production of TNF‐α and IFN‐γ. In addition, there may be a form of crosstalk between CCR5 and CCR2. Inconsistently, infusion of CCR5^−/−^ Tregs into lethally irradiated mice significantly increased the infiltration of CD4^+^ and CD8^+^ T cells into the liver, resulting in earlier and more severe GVHD.

**Conclusion:**

This review indicates that CCR5 plays an important role in pathogenesis and development of acute GVHD. Elucidating its role in different immune cells will aid the development of targeted therapeutic treatments.

## Graphical abstract

Our previous studies demonstrate that CCR5 blockade could inhibit donor T cells migration and recruitment toward target organs, reduce the amount of donor T cells in absolute numbers, be capable of slightly suppressing dendritic cells maturation, and reduce the percentage of Th1 and Th17 subsets. CCR5 blockade also inhibits internalization and activation of chemokines, inhibits proliferation and chemotaxis of T cells, and decreases the production of TNF‐α and IFN‐γ. In addition, there may be a form of crosstalk between CCR5 and CCR2.

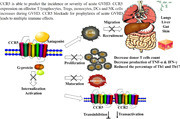

## 1 INTRODUCTION

The C–C chemokine receptor 5 (CCR5) belongs to the superfamily of seven transmembrane G‐protein coupled receptors (GPCRs). It interacts with multiple chemokine ligands that mediate the migration and function of T lymphocytes, monocytes, macrophages, dendritic cells (DCs), and natural killer (NK) cells to sites of inflammation.^1^, ^2^, ^3^ When bound to specific ligands, the receptors can be subsequently internalized, impairing their binding capacity. Once internalized, GPCRs tend to recycle to the cell surface. Most ligands activate more than one chemokine receptor, whereas CCR5 is able to bind several ligands (mainly CCL3‐5). After activation with small chemokines, GPCRs initiate rapidly phosphorylated at threonine and serine residues leading to a multitude of cellular signal transduction pathways.^4^, ^5^, ^6^, ^7^ CCR5 and its ligands' interactions direct the migration of effector cells to target tissues and regulate their activation during inflammation. CCR5 is expressed on a broad range of cells, including microglia, astrocytes, neurons, fibroblasts, and also on the epithelium, endothelium, and vascular smooth muscle.^8^, ^9^ Hence, CCR5 has been implicated in the pathophysiology of a number of diseases.

CCR5 is the main coreceptor for macrophage‐tropic strains of the human immunodeficiency virus (HIV), which allows, along with CD4, binding of the viral particles to the cell surface through its envelope protein gp120, triggering the membrane fusion reaction.^10^, ^11^ Published studies show that CCR5 not only participates in the pathogenesis of AIDS, atherosclerosis, rheumatoid arthritis, graft rejection, and neurodegenerative diseases but also plays an important role in the development and progression of acute graft‐versus‐host disease (GVHD) following allogeneic hematopoietic cell transplantation (AHCT).^1^, ^2^, ^10^, ^11^, ^12^, ^13^ CCR5 is able to predict the incidence or severity of acute GVHD, particularly together with other chemokines (e.g., IL‐6, IL‐8, RANTES). Current therapies for GVHD target CCRs may antagonize the function of T cells and affect immune responses through cytokine regulatory networks.^14^, ^15^, ^16^ CCR5 blockade inhibits donor T cell migration, reduces the amount of donor T cells, is capable of suppressing DC maturation, and reduces the percentage of Th1 and Th17 subsets. CCR5 blockade also inhibits activation, proliferation and chemotaxis of T cells, and decreases the production of TNF‐α and IFN‐γ. Herein, we will review the latest studies and findings to investigate the relationship between CCR5 and acute GVHD, and the multiple immune effects of targeting CCR5.

## 2 STRUCTURE, INTERNALIZATION, AND FUNCTION OF CCR5

The location of the CCR5 gene and its amino acid sequence was firstly discovered and elucidated in 1996.^17^ The position of CCR5 was mapped relative to the other CCR genes at this locus on chromosome 3p21, approximately 1.9 kb in length, contains four exons and two introns. CCR5 is closely linked in a 350 kb region with CCR2, CCR3, and CCR1. Significantly, CCR5 is located downstream of CCR2, the gene to which it is most similar, and the two are closest apart, with an open reading frame separated by only about 17.5 kb. CCR5 has two independent promoters, which can be recognized and bound by a number of transcription factors (AP‐1, GATA‐2, STAT3, NF‐κB) to regulate the transcriptional activity. Alternative splicing complex and variable transcription initiation sites cause the unique spliceosome structure of CCR5. Only two splices were identified so far: *CCR5A* and *CCR5B*, with the difference mainly in the 5′‐untranslated region. The transcription product of the CCR5 gene is about 3.5 kb in length and encodes a 352‐amino acid transmembrane protein with a molecular weight of 40.6 kDa. The N‐terminal region of CCR5 lacks glycosylation sites, which is different from most GPCRs. Disulfide bonds are formed between the cysteines of the first and second extracellular loops of CCR5 to ensure their stability.

Receptor expression at the surface is a balance between the rate of replacement and internalization (recycling and new synthesis). Ligand binding to CCR5 is induced to undergo a major conformational change. There are two major routes when CCR5 binds to its specific ligands.^18^, ^19^, ^20^ The first pathway includes the binding of arrestin to CCR5, which leads to the movement of CCR5 to clathrin‐coated pits and internalization. The second pathway depends on caveolae, not clathrin‐coated pits. The clathrin‐coated pits‐dependent pathway is the major entry system into cells, which is also considered a default system for degradation and recycling. Arrestin‐2 bond to the phosphorylated receptor, in turn, initiates the internalization through combination with clathrin. The receptor‐arrestin‐2 complex is subsequently sequestered in clathrin‐coated pits. The clathrin‐coated pits are pinched off to become vesicles by the action of dynamin. The vesicle fusion processes are involved in the trafficking of vesicles from early endosomes to late endosomes, finally to the lysosomes.^5^, ^21^

CCR5 is mainly expressed on monocytes, DCs, activated T cells, macrophages, and NK cells. The presence of CCR5 on regulatory T cell subsets (Tregs) suggests that Tregs are able to enter inflamed tissues. Its ligands include MIP‐1α (CCL3), MIP‐1β (CCL4), RANTES (CCL5), MCP‐2 (CCL8), and MCP‐1 (CCL2), MCP‐3 (CCL7, antagonist), MCP‐4 (CCL13), and eotaxin (eotaxin‐1/CCL11, eotaxin‐2/CCL24, eotaxin‐3/CCL26, agonists of CCR5).^22^, ^23^ CCL3, CCL4, and CCL5 are able to effectively bind to CCR5 and belong to complete agonists. By contrast, CCL7, CCL8, and CCL13 have weak binding capacity and activity. Interestingly, the binding of MCP‐3/CCL7 to CCR5 is unable to mediate the transmission of signals and is a natural antagonist.^24^

CCR5 is frequently expressed at the surface of leukocyte populations as monomers, dimers, or oligomers.^25^ Heteromerization has been demonstrated between CCR2 and CCR5 or CXCR4, which may result from a high degree of structural homology.^26^, ^27^, ^28^ After the heterodimer binds to the ligand, its function and signal transduction are still controversial. It is indicated that the CCR5/CCR2 dimer formation was ligand‐dependent. Whereas, previous studies demonstrated dimers formation before transport to the endoplasmic reticulum, which is ligand‐independent. El‐Asmar et al.^29^ found that the formation of CCR5/CCR2 homo‐ or heterodimers was random and ligand‐independent. Sohy et al.^30^ first demonstrated the presence of CCR5/CCR2/CXCR4 heterotrimers, while antagonists of one receptor can cross‐inhibit other receptors in the oligomer, such as cenicriviroc, an antagonist of CCR2 and CCR5, which can inhibit cell recruitment mediated by the CXCR4 agonist SDF‐1α.

CCR5 acts as a coreceptor for HIV‐l and involves in the entry process.^10^, ^11^ A 32 base pair deletion of the CCR5 gene (*CCR5‐delta32*) located on chromosome 3 results in a nonfunctional protein. It is thought that this mutation causes an alteration in T lymphocytes' response to inflammation. The presence of the *CCR5‐delta32* allele in recipients of allografts is considered to be a protective factor associated with a decreased risk of GVHD and graft rejection.^31^, ^32^ CCR5 and its ligands mediate recruitment of immune cells, which not only participate in the pathogenesis of a variety of inflammatory diseases, but also in pathophysiological processes such as tissue injury, angiogenesis, and hematopoiesis.

## 3 CCR5 AND ACUTE GVHD

CCR5 and its natural ligands have been implicated in the pathogenesis of GVHD. Acute GVHD is one of the major causes of death following AHCT, which is a T‐cell‐mediated inflammatory disease.^13^, ^33^, ^34^, ^35^ In Billingham's^36^ classic description of the elements required for the development of acute GVHD, three conditions were required: (1) the host must be incapable of rejecting the graft, (2) the graft must contain immunocompetent cells, and (3) there must be incompatibilities in transplantation antigens between donor and host. However, a fourth requirement must be met, which was first proposed as a corollary to Billingham's criteria: the effector cells must migrate to the target tissues.^37^, ^38^ Thus, the expression of chemokine receptors on T lymphocytes and the level of chemokines in the environment of the target tissues after AHCT determine whether effector T cells migrate and recruit to target organs, thereby triggering GVHD injury. The activation, proliferation, differentiation, and migration of donor T lymphocytes to target organs, together with Tregs that induce immune tolerance, play important roles in the pathogenesis of GVHD. Previous reports indicate that CCR5 is critical for lymphocyte recruitment to tissues involved in GVHD.^39^, ^40^, ^41^, ^42^

### 3.1 Increased CCR5 expression during acute GVHD

CCR5 expression on effector T lymphocytes, Tregs, monocytes, DCs, and NK cells increases during GVHD. Published animal and clinical studies demonstrate that CCR5 expression is elevated when acute GVHD occurs. Early results indicated that the CCR5 gene‐expression levels in patients increased a few days before acute GVHD was diagnosed clinically.^43^ Additionally, within a few days after transplantation, the concentration of CCL3, CCL4, and CCL5 in secondary lymphoid tissues all increased. CCR5 is essential for the recruitment of Tregs to GVHD target tissues as a prerequisite for their suppressive function on alloreactive T cells. The confirmative protein analyses demonstrated a significantly higher gene expression of CCR5 in Tregs of immune tolerant patients.^44^

CCR5 is also important for the development of GVHD in different target tissues such as skin, liver, and gut. The maximum percentage of CCR5^+^CD16^+^ DCs, at any time after transplantation, correlated with development of cutaneous GVHD. The expression of CCL5 in the skin revealed an increasing trend 2 weeks following AHCT while infiltrating cells were dominantly CCR5 positive.^45^ The skin pathological examination of patients who developed acute GVHD showed that infiltrating CD4^+^ and CD8^+^ T cells highly expressed CCR5.^46^ In a murine model, CCR5^+^CD8^+^ T cells infiltrated the liver, while CCL3 concentration increased in intralobular biliary epithelial cells, endothelial cells, and macrophages, resulting in liver damage. CCL5 levels were significantly increased in the liver during acute GVHD.^47^, ^48^ CCR5 was essential for migration of CD8^+^ T cells into Peyer's patches and development of intestinal GVHD.^49^ These results indicate that upregulation of the CCR5 gene and surface expression intimately relates to the development of acute GVHD.

### 3.2 Inhibition of CCR5 and acute GVHD

A significant association between the common CCR5 haplotype (H1/H1) and the advantage of overall survival in recipients of AHCT has been found. The recipients with the *CCR5‐delta32* allele had a lower incidence of grade I–II acute GVHD. If the donor is *CCR5‐delta32* homozygous, none of the GVHD manifestations is observed after transplantation.^50^ Hence, the *CCR5‐delta32* mutation is an independent protective factor that predicts a lower incidence of acute GVHD. CCR5 genotyping may be a new diagnostic and prognostic strategy for therapy optimization.

CCR5 is selectively induced in the effector phase, primarily on CD8^+^ T cells, and the migration of CCR5^+^ cells may depend on MIP‐1α. Therefore, anti‐CCR5 antibody treatment reduced liver injury as a result of the depletion of CCR5^+^ cells in the liver.^51^ The addition of total body irradiation‐induced higher expression of CCR5 on human CD4^+^ and CD8^+^ T cells promoted their migration and proliferation in organs, resulting in more severe tissue damage.^52^ CCR5 is essential for Tregs, and CCR5^−/−^ Tregs are able to inhibit the proliferation of T cells both in vitro and in vivo. Wysocki et al.^42^ demonstrated that irradiated mice transferred with CCR5^−/−^ T cells had an earlier time to onset and worsening GVHD compared to nonirradiated recipients due to higher expression of pro‐inflammatory chemokines such as CXCL10 and CXCL11 which were responsible for CCR5^−/−^ T cell migration to target tissues. In a murine GVHD model, mice cotransplanted with CCR5^−/−^ Tregs, had increased GVHD scores and decreased survival.^47^ Furthermore, infusion of CCR5^−/−^ Tregs into lethally irradiated mice significantly increased the infiltration of CD4^+^ and CD8^+^ T cells into the liver, resulting in earlier and more severe GVHD.^53^ The controversial results suggest that CCR5 knockout in donor T cells is influenced by various factors such as conditioning regimen, and its role needs further investigation.

Our previous study indicated that reduction of CCL5 level, as well as downregulation of CCR5, were observed in mesenchymal stem cell (MSC) cotransplantation mice. CCL5 is positively associated with the development and severity of idiopathic pneumonia syndrome. After lethal irradiation, compared to nontransplanted mice, MSC‐transplanted mice exhibited significantly increased survival, as well as higher Treg percentage, reduced IFN‐γ, CXCR3 and CCR5 downregulation, and CCR7 upregulation.^54^ Different donors responded to recombinant human granulocyte colony‐stimulating factor (rhG‐CSF) mobilization differently being downregulated or upregulated in varying degrees. There were great disparities in expression changes of CCR5 among the donors after mobilization. But rhG‐CSF definitely affects the migration and recruitment of T cells to target organs in vivo, thereby reducing the incidence of acute GVHD.^55^

**Figure 1:**
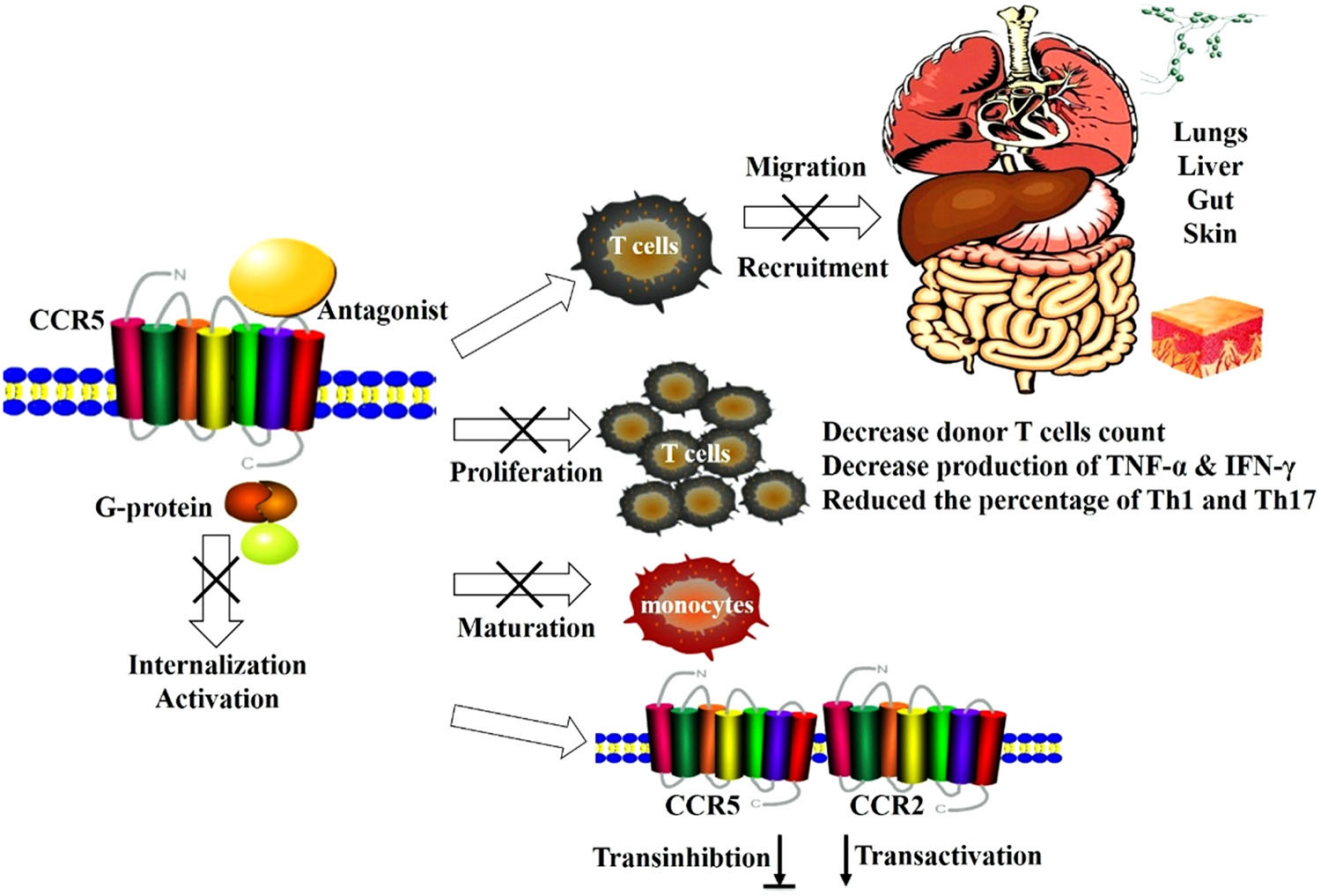
The multiple immune effects of targeting CCR5 for prophylaxis of GVHD. CCR5 blockade inhibits donor T cell migration and recruitment toward target organs, reduces the absolute numbers of donor T cells, is capable of slightly suppressing DC maturation, and reduces the percentage of Th1 and Th17 subsets. CCR5 blockade also inhibits the internalization and activation of chemokines, proliferation, and chemotaxis of T cells, and decreases the production of TNF‐α and IFN‐γ. There may be a form of crosstalk between CCR5 and CCR2. CCR5, C–C chemokine receptor 5; GVHD, graft‐versus‐host disease.

CCR5 blockade significantly reduces the severity of acute GVHD.^56^ Maraviroc, a small molecule CCR5 antagonist, significantly alleviated the degree of visceral injuries and prolonged survival time. In clinical studies, maraviroc did not affect immune cell reconstitution, infection or relapse rates. This suggests that CCR5 blockade does not affect graft‐versus‐leukemia or graft‐versus‐infection.^56^, ^57^, ^58^ Whereas Bolaños‐Meade et al. reports that the short course of maraviroc has no benefit in terms of GVHD‐free relapse‐free survival compared to controls. These data can be consistent with those obtained in mouse GVHD models showing that the role of CCR5 in acute GVHD may depend on the intensity of the conditioning regimen.^42^, ^59^ CCR5 blockade decreases peripheral T‐cell activation, gut GVHD biomarkers (e.g., serum reg3a, CD146), and acute GVHD incidence in allogeneic HCT recipients. The level of CCR5, as well as CCL5, and pro‐inflammatory cytokines in the liver and intestines were inhibited after cenicriviroc treatment.^60^, ^61^, ^62^, ^63^ Our murine model studies demonstrated that CCR5 blockade by maraviroc attenuates liver GVHD by impairing T cells function. The mechanisms mainly include that maraviroc could inhibit donor T cell migration to target organs, reduce the absolute numbers of donor T cells, be capable of slightly suppressing DC maturation, and reduce the percentage of Th1 and Th17 subsets. CCR5 blockade also inhibits CCR5 and CCR2 internalization induced by CCL5 and CCL2, T cell chemotaxis, and decreases the production of TNF‐α and IFN‐γ (Figure 1).^64^, ^65^, ^66^ These results suggest that the immunoregulation network is complicated and there may be a form of crosstalk between CCR5 and CCR2. One major challenge in targeting ligand–receptor interactions is the large redundancy of the chemokines system and the possibility of counterregulatory activation of alternative pathways.

## 4 DISCUSSION

To conclude, a better understanding of the pathophysiology of GVHD has led to the development of novel strategies to prevent GVHD. CCR5 is a marker for GVHD effector cells, whereas CCR5 expression is elevated when acute GVHD occurs. CCR5 is able to predict the incidence or severity of acute GVHD, particularly together with other chemokines. The interaction between CCR5 and its ligands is a hotspot in current medical research. Our murine model studies indicate that CCR5 blockade not only inhibits donor T cell migration and recruitment, reduces the absolute numbers of donor T cells, is capable of slightly suppressing DC maturation, but also decreases Th1 and Th17 subsets. Blocking CCR5 inhibits the internalization and activation of chemokines, proliferation, and chemotaxis of T cells, and reduces the production of TNF‐α and IFN‐γ. In addition, there may be a form of crosstalk between CCR5 and CCR2. Nevertheless, the specific role of CCR5 on different immune cells is very complex, and heterogeneity between individuals is also evident. Ongoing clinical studies and laboratory investigations will add valuable information and may change our current perception.

## CONFLICT OF INTEREST

The authors declare no conflict of interest.

## Data Availability

The data that support the findings of this study are available from the corresponding author upon reasonable request.
